# Minor changes in fibre intake in the UK population between 2008/2009 and 2016/2017

**DOI:** 10.1038/s41430-021-00933-2

**Published:** 2021-05-13

**Authors:** Mathilde Gressier, Gary Frost

**Affiliations:** 1 Section for Nutrition Research, Department of Metabolism, Digestion and Reproduction, Faculty of Medicine, Imperial College London, London, UK; 2 Centre for Health Economics & Policy Innovation, Department of Economics & Public Policy, Imperial College Business School, Imperial College London, London, UK

**Keywords:** Risk factors, Biomarkers

## Abstract

The benefits of increasing populations’ and individuals’ fibre intake on non-communicable disease risk have been known and promoted for decades in the UK and in the world. Public health campaigns, including dietary recommendations, called populations to increase their consumption of whole grains, fruits and vegetables, while manufacturers increased the fibre content of their products. In particular, the SACN report in 2015 highlighted the importance of fibres for the UK population. We analysed trends in fibre consumption for the whole population, by age group and gender using the UK National Diet and Nutrition Survey from 2008/09 to 2016/17. We investigated changes in total fibre intake and calculated the contribution to fibre intake and time trends from each food group. We compared the fibre content of food groups between 2008/09 and 2016/17. We found that fibre intake remained fairly stable. While the fibre content of some cereal-based products increased, it decreased for potato-based products. All age groups derived increasing fibre from pasta and other cereal-based products, and decreasing fibre from potato products. Adults, but not children or adolescents derived more fibre from vegetables. This resulted in an increase in fibre intake in adults, but not in children or adolescents.

## Introduction

Dietary fibres are component of plant-based foods that are not digested or absorbed in the small intestine [1] but fermented by micro-organisms in the gut. Their consumption is linked to various health benefits, including reducing the risk of cardiovascular diseases or type 2 diabetes and bowel cancer [2]. Intake of dietary fibres is consistently lower than recommended targets in all regions of the world. Intake is lowest in Asia (~8–10 g/day) and highest in Oceania and western and eastern sub-Saharan Africa (~16–20 g/day) [3]. The inadequate consumption of fibres was estimated to be responsible for about 100,000 deaths in 2017 globally [3]. In the UK, the importance of fibres in the diet was emphasized in the 2015 report of the Scientific Advisory Committee on Nutrition (SACN, [1]). The report recommends a daily fibre intake of 30 g/day or more for adults, a level from which the greatest health benefits were observed [1]. This message was promoted by various organizations promoting healthy diets, which designed new messages and campaigns to raise population awareness about the benefit of an adequate fibre intake. Most of these messages, such as the messages from the NHS, the British Heart Foundation or the British Nutrition Foundation, put emphasis on swapping refined grains to whole grains, as fibre and other healthful minerals are lost during the refinement of grains. The message to eat five fruits and vegetables a day was also reinforced [4–6]. In addition, food manufacturers formulate foods with increasing levels of fibres to improve their nutrient profile [7]. One strategy is to substitute refined grains with whole grains or to launch new products with whole grains. The focus on whole grains started in the 2000s, with the introduction of a whole grain guideline in the US dietary guidelines [8], or the launch of the Danish Whole Grain Partnership in 2007 [9]. This increased focus on whole grains incentivised manufacturers to reformulate and formulate new products with whole grains [8]. There is currently limited evidence on the effect of this increased focus on dietary fibres in public health messages, policies, and food product innovations on fibre intake in the UK. The National Diet and Nutrition Survey (NDNS) rolling programme between 2008/09 and 2016/17 showed an increased intake of total fibres (of 0.3 g/year) [10], however, this figure does not capture reformulation, as the total fibre content of NDNS foods were added retrospectively in 2015/2016 [10]. In the US, fibre intake increased in children, adolescents, and adults aged over 51 years between 2001 and 2010 [11]. In this study, we investigated the evolution of fibre intake in the UK population between 2008/09 and 2016/17, with the SCAN recommendation published in 2015. Also, we investigated reformulation of food categories and possible changes in the food sources of fibres.

## Materials and methods

Daily food intakes of the UK population were obtained from the NDNS from 2008/09 to 2016/17 [12]. We derived the nutrient composition of each food code (i.e. food reported in the survey) for each survey year from the Nutrient Databank, a regularly updated food composition table created from the McCance & Widdowson table, made to match each survey year of the NDNS [12]. Food products were grouped into 17 food groups (listed in Table 1) defined in the McCance & Widdowson table, separating potato-based products from vegetables to analyse these two food groups separately. The mean non-starch polysaccharide (NSP) fibre content of each food group (and of all foods) was calculated by averaging the fibre content of all foods appearing in each food group each year of the survey. Mean NSP fibre contents in years 2008/09 and 2016/17 were compared with the non-parametric Mann–Whitney *U*-test. To test changes in fibre content of paired food products (i.e. foods recorded in both surveys from 2008/09 and 2016/17), Wilcoxon signed-rank tests were used. Both tests were done first across all foods, then for the foods belonging to each food group.

**Table 1:** Number and NSP fibre content of food products recorded in NDNS in 2008/09 and 2016/17.

	No. of products 2008/09	No. of products 2016/17	No. of paired products	Median 2008/09	Mean ± SD 2008/09	Median 2016/17	Mean ± SD 2016/17	*p*-val Mann–Whitney-*U*	*p*-val paired Wilcoxon
Nuts & seeds	40	41	40	5.5	5.4 ± 2.7	5.4	5.7 ± 3.6	0.8984	0.5839
Breakfast cereals	130	176	123	4.3	5.3 ± 4.7	4.9	5.3 ± 4.1	0.4790	0.3904
Bread	106	121	103	3.15	3.6 ± 1.8	3.7	3.8 ± 1.9	0.3414	**0.0039**
Pasta, Rice & other cereals	185	194	183	1.3	2.7 ± 4.2	1.3	2.6 ± 4.1	0.7532	0.1421
Sauces, soups and condiments	297	355	295	0.5	2.5 ± 6.4	0.7	2.9 ± 6.9	0.0721	0.0847
Vegetables	394	447	378	1.9	2.5 ± 2.3	1.9	2.5 ± 2.2	0.4929	0.0759
Fruit	209	257	208	1.3	2.1 ± 2.8	1.3	2.0 ± 2.7	0.9431	**0.0002**
Biscuits, pastries and puddings	467	501	461	1.4	1.8 ± 1.8	1.6	2.0 ± 2.4	0.0773	**0.0096**
Potato-products	138	139	129	1.5	1.6 ± 0.7	1.2	1.3 ± 0.7	**0.0000**	**0.0000**
Sugars, preserves & snacks	212	224	205	0.35	1.2 ± 1.9	0.7	1.3 ± 2.0	0.0712	0.0602
Meat and meat products	692	696	688	0.4	0.6 ± 0.6	0.5	0.6 ± 0.6	0.1564	**0.0000**
Supplements	301	594	294	0	0.3 ± 1.2	0	0.4 ± 4.2	0.3023	0.9165
Eggs	78	80	78	0	0.3 ± 0.5	0	0.3 ± 0.5	0.6247	0.0830
Fish & fish products	215	221	212	0	0.3 ± 0.4	0	0.3 ± 0.4	0.9264	0.4151
Milk & milk products	267	342	246	0	0.2 ± 0.5	0	0.3 ± 0.7	0.0872	**0.0047**
Fats and oils	55	71	51	0	0 ± 0.1	0	0 ± 0.1	0.4806	1.0000
Beverages	229	278	221	0	0 ± 0	0	0 ± 0.1	**0.0049**	0.1198
All foods	4015	4737	3915	0.7	1.5 ± 2.8	0.7	1.5 ± 3.3	0.6502	**0.0045**

Mean (±SD) and median NSP fibre content of foods are calculated from all foods recorded in the survey-year. Changes in the distribution of fibre content were tested using Mann–Whitney *U*-test on all foods reported in each survey year. Paired Wilcoxon-rank sum test was used for food items recorded in the survey both years. Bold values show a significant test at 5%.

We estimated the daily mean NSP fibre intake of the UK population in each of the 9 years of the survey. Trends in intake were estimated using linear regression, adjusted for demographic characteristics (age group, gender, BMI, ethnicity and equivalised income) and total energy intake. We added interaction terms between survey year and age group or sex to test the differential change by age or sex. We identified the food sources of NSP fibres for the UK population by averaging the contribution of each food group to participants’ daily fibre intake (including consumers and non-consumers for each food group). Trends in the contribution of each food group to total fibre intake were estimated using linear regression, adjusted for the demographic and energy intake variables defined above. To take into account the sampling procedure of the NDNS and get estimates representative of the UK population, sampling weights were used in each procedure.

## Results

Overall, there was no change in the mean NSP fibre content of all foods between 2008/09 and 2016/17 (mean content of 1.5 g/100 g in the two years, non-significant Mann–Whitney *U* test, Table 1). Nonetheless, there was a change in the composition of paired products (present in both 2008/09 and 2016/17, with a *p*-value = 0.0045 (Wilcoxon test)) (Table 1). The mean fibre content of some cereal-based products increased (such as bread: mean content of 3.6 ± 1.8 g/100 g in 2008/09, 3.8 ± 1.9 g/100 g in 2016/17), while one of the potato products decreased (mean content of 1.6 ± 0.7 g/100 g in 2008/09, 1.3 ± 0.7 g/100 g in 2016/17) (Table 1).

**Fig. 1:**
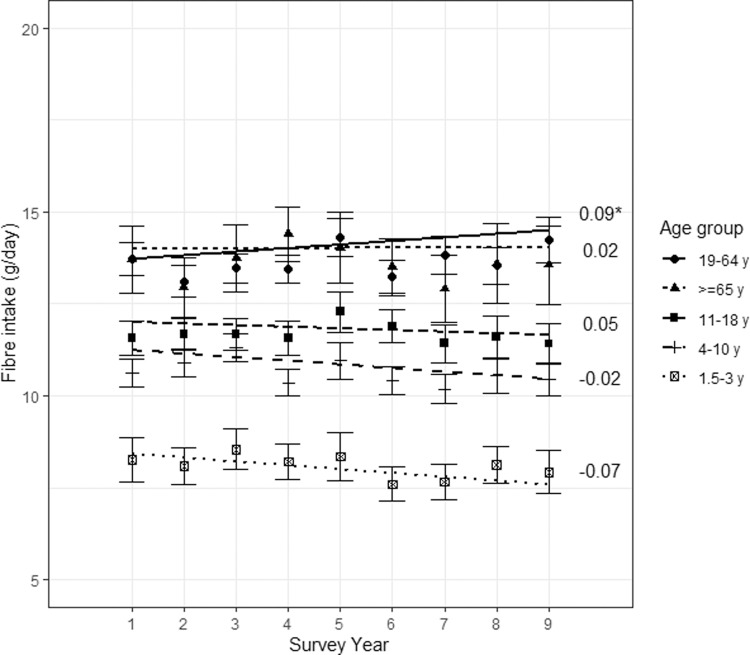
Trend in mean fibre intake of the UK population between 2008/09 and 2016/17, by age group. The graph shows mean fibre intake and 95% confidence intervals for each age group and each survey year of the NDNS. Trendlines were plotted for each age group. Corresponding estimates for the time trends are shown on the right of the graph (corresponding to the interaction term between survey year and age group). Asterisks (*) indicates a significant estimate at a 5% level.

For the whole population, there was a marginal but significant increase in fibre intake (adjusted trend: +0.06 g/year, *p*-value = 0.032, adjusted for age group, sex, energy intake, BMI, ethnicity and equivalised income). However, after adding an interaction between age group and survey year, this significant increase was only found in adults (Fig. 1). Adding an interaction between sex and survey year revealed a significant increase in fibre intake only in males (adjusted trend: +0.09 g/day/year, *p* = 0.041), but not in females. Similar results were obtained when the regressed term was fibre intake for 10 MJ (the adjusted trend for males: +0.10 g/10 MJ/year, *p* = 0.026).

**Fig. 2:**
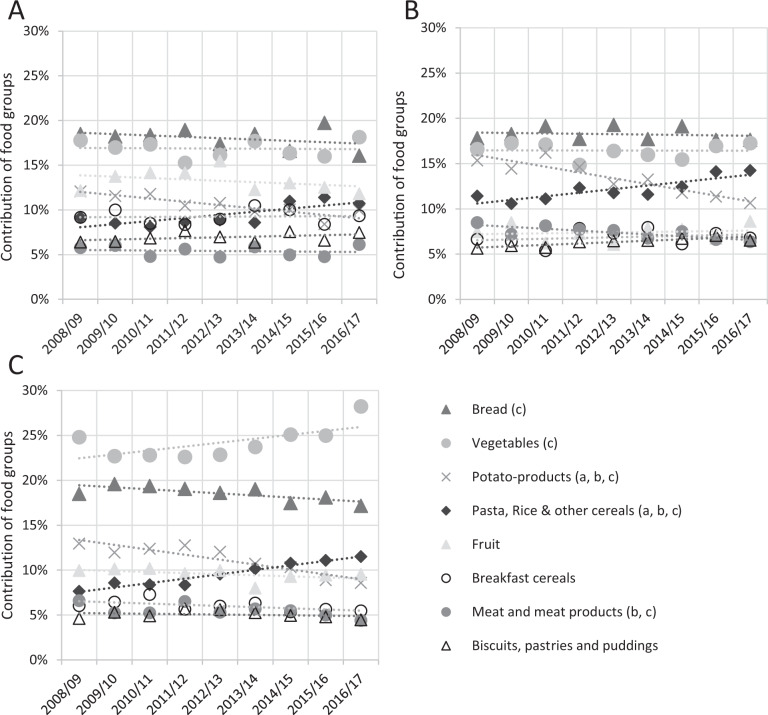
Mean contribution of each food group to non-starch polysaccharide (NSP) intake between 2008/09 (Year 1) and 2016/17 (Year 9) by age group. Contributions for children aged 4--10 years and shown in (**A**), for adolescents aged 11–18 years in (**B**) and adults aged 19–64 years old (**C**). Letters ‘a’, ‘b', or ‘c’ in the legend indicates a significant time trend (significant effect of year on mean fibre intake), for the Contributions for children aged 4--10 years and shown in (A), for adolescents aged 11–18 years in (B) and adults aged 19–64 years old (C).

Cereal-based products (bread, pasta, rice and other cereals, breakfast cereals, biscuits, pastries and puddings) were the first contributor to NSP fibres intake in children, adolescents and adults. Bread alone corresponded to ~20% of fibre intake in children, adolescents and adults (Fig. 2). The vegetable was the second contributor, followed by potato products and fruits. For all age groups, the contribution of potato products to NSP fibres decreased between 2006/07 and 2016/17, while the contribution from pasta, rice and other cereals increased. In adults, the contribution of vegetables increased (Fig. 2C), but not for children (Fig. 2A) or adolescents (Fig. 2B).

## Discussion

Although multiple initiatives were implemented to increase fibre intake in the UK population [1], intake remained fairly stable. A small increase of 0.09 g/day per survey year was found in adults, small in the comparison to the gap between estimated mean fibre intake in adults (14.9 g/day, SE = 0.5) and recommendations (30 g/day). Several reasons can explain this resistance to add fibre-rich food to diets. First, the sensory profile of whole-grain products is not accepted by some consumers, unlikely to switch from refined grain- to whole grain products [13]. Also, some consumers link starchy foods to weight gain or digestion discomfort, hence do not want to increase their intake of fibre-containing starchy foods [14].

For all age groups, there was a shift from fibres from potato products to pasta, rice, and other cereals. Beyond fibre intake, this increased consumption of cereal-based products can have a varying impact on other nutrients if the shift was in favour of whole or refined grain products. Refined grain products have fewer vitamins and minerals than whole-grain products; despite refined flour being enriched in vitamins and minerals, wholemeal bread remains a better source of vitamins and minerals than white bread [15].

The evaluation of trends in intake from NDNS by Public Health England (PHE) found increased intake of total fibres (without considering the effect of reformulation) (of 0.3 g/year) between 2006/07 and 2016/17 [10], while we found an increase in NSP fibres (including the effect of reformulation) of 0.06 g/year. The difference between these results can come from the increased consumption of foods containing non-NSP fibres such as resistant starch, inulin or polydextrose. These fibres are increasingly used as a food supplement or added to food products to improve their sensory or nutritional characteristics [7]. Thus, our estimates of reformulation based on NSP fibres are likely to underestimate the change in total fibres. The NDNS report also found a significant increase in total fibre intake for adult men but not for women.

In conclusion, this study shows that fibre intake remained fairly constant in the 2010s, despite information campaigns about how to increase one’s fibre intake, and actions from manufacturers to increase the fibre content of cereal-based products. The source of fibre changed, with adults deriving more fibre from vegetables and less from potato-based products. This could be the effect of public health campaigns highlighting the importance of an adequate vegetable intake. However, this favourable increase in fibre from vegetables was not observed in children and adolescents. Children and adolescents derived more fibre from pasta and other cereal-based products, but this was not enough to be translated into a change in fibre intake. Cereal-based products have a higher fibre density than vegetables, but vegetables are nutrient-dense and have a low energy density, making them essential in children diets. Although the reformulation of cereal-based products to increase their fibre content and their promotion is laudable to improve fibre intake of the population, efforts should also focus on increasing vegetable consumption in children and adolescents.
